# Michael Ter-Avanesyan (1949-2018) - life in science

**DOI:** 10.1080/19336896.2019.1567201

**Published:** 2019-01-11

**Authors:** Vitaly V. Kushnirov

**Affiliations:** Research Center of Biotechnology of Russian Academy of Sciences, A.N. Bach Institute of Biochemistry, Moscow, Russia

**Keywords:** Michael Ter-Avanesyan, Sup35, prion, amyloid, translation termination

## Abstract

This commentary describes scientific path and accomplishments of our late colleague, Prof. Michael D. Ter-Avanesyan, who made several seminal contributions into prion research.

Michael Davidovich Ter-Avanesyan (Figure 1) studied and then started his career (1971) at the Department of Genetics of the St. Petersburg State University. In 1983, as a productive promising scientist, he was offered an independent position to continue his studies at the Cardiology Research Center, Moscow. In 1988, Michael’s group became a laboratory, named Laboratory of Molecular Genetics, and in 2011, this laboratory moved from the Cardiology Center to the Bach Institute of Biochemistry, Russian Academy of Sciences.

**Figure 1.**
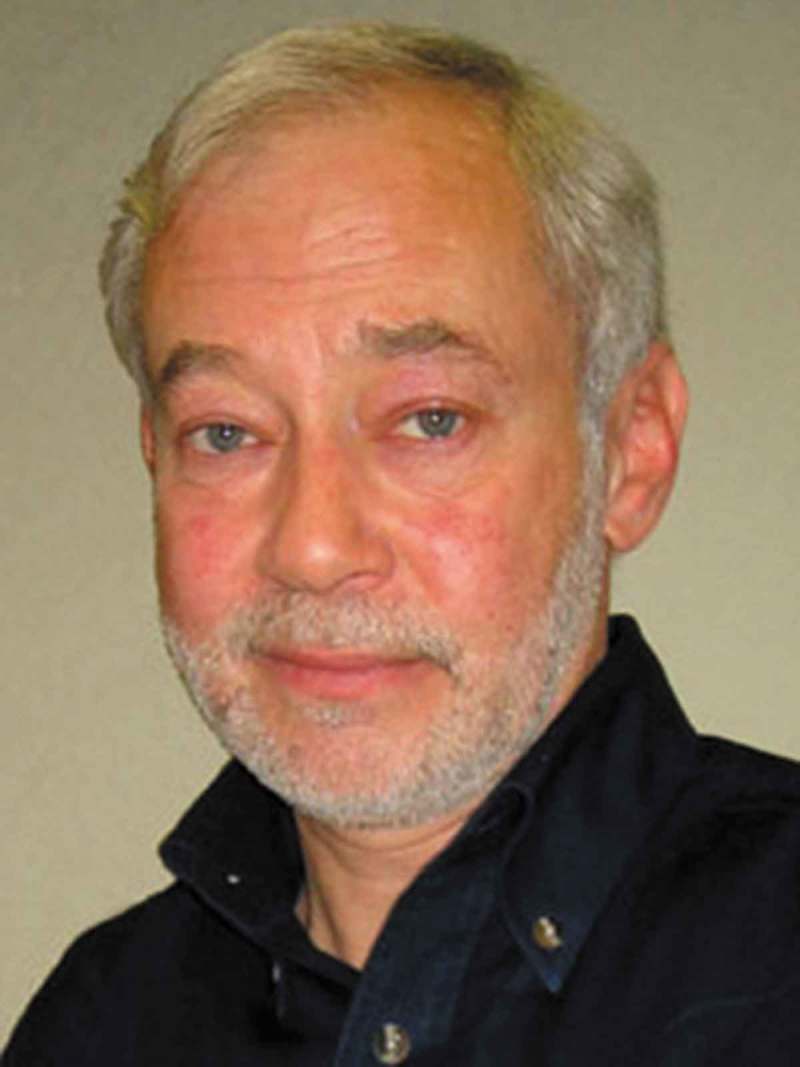
Michael Ter-Avanesyan in 2015.

I joined Michael’s group in 1986. At that time, Cardiology Center looked like a fairy tale compared to other research institutions of the USSR, being equipped in accordance to the highest world standards, since it was built for and managed by the personal cardiologist of the Soviet leader Leonid Brezhnev, academician Evgeny Chazov. However, the tale did not last long, because soon the Soviet Union experienced a deep crisis and collapsed, and funding was reduced to a minimum.

Michael studied yeast omnipotent nonsense suppressor genes *SUP35* and *SUP45* at that period. The accompanying article by D.A. Gordenin and L.N. Mironova [1] describes how his interest in this area has evolved. After performing a lot of classic genetics studies on these genes, Michael started switching to molecular studies. At first, it was unclear what are the functions of these genes. However, many interesting observations were made, including that mutations in these genes can cause respiratory deficiency [2] and that growth of some mutants, paradoxically, requires the presence of translational inhibitor, cycloheximide [3]. Meanwhile, Michael with colleagues had cloned and sequenced the *SUP35* gene, and found that the protein encoded by this gene is composed of three very different regions. The С-terminal region was essential for viability and similar to translation elongation factor eEF-1A, while the N-terminal and middle regions were dispensable [4,5]. Also, the N-terminal region was suspiciously rich in glutamine and tyrosine. In the next round of *SUP35* studies, Michael found that it is tightly related to the mysterious non-Mendelian genetic determinant [*PSI*^+^] and that the N-terminal glutamine-rich region is necessary and sufficient for the maintenance of this determinant [6]. Somewhat earlier, it was shown that [*PSI*^+^] is lethal in combination with increased copy number of *SUP35* or in diploid strains lacking one of *SUP45* copies [7,8].

In 1994 and 1995, the studied field exploded with major discoveries. Lyudmila Frolova with coauthors showed *in vitro* that human and Xenopus homologs of Sup45 are the eukaryotic translation termination factors, designated as eRF1 family [9]. Next year, Galina Zhouravleva and coauthors extended this finding to Sup35 (eRF3) [10], while Michael’s lab, together with the lab of Mick Tuite, provided genetic evidence that Sup35 cooperates with Sup45 in yeast translation termination [11]. But the most interesting findings were related to the prion nature of Sup35, which was proposed by Reed Wickner based on the genetic data [12]. Michael’s laboratory, slightly ahead of the lab of Susan Lindquist [13], supported this hypothesis by biochemical data by showing that in [*PSI^+^*] cells Sup35 protein is aggregated through its N-terminal domain and these aggregates can append new molecules of soluble Sup35 [14]. Developing this work further, Michael showed that the extracts of [*PSI^+^*] cells can nucleate aggregation of Sup35 protein *in vitro*, and this aggregation can be cyclically reproduced with high efficiency and through numerous sequential cycles [15]. This paper provided the most convincing support for the prion hypothesis in general at that time, and served as a basis for the development of the Protein Misfolding Cyclic Amplification (PMCA) techniques for mammalian proteins by other researchers in future [16]. The next important question was how prions multiply *in vivo*. The key player here proved to be the Hsp104 chaperone, whose connection to yeast prions was discovered by Yury Chernoff in 1995. The effects of Hsp104 were paradoxical: Hsp104 was strictly required for [*PSI*^+^] propagation, but excess of Hsp104 was also detrimental for [*PSI*^+^] [17]. Together with Michael, we offered a simple explanation for the mechanism of Hsp104 action [14,18]. This explanation combined two additional findings. First, Parsell et al. showed that Hsp104 extracts protein molecules from large heat-denatured aggregates [19], and second, Glover and King showed that Sup35 can form amyloid fibrils *in vitro* [20,21]. However, what would happen when Hsp104 would extract a molecule from such a fibril? – Logically, the fibril will be split in two parts, both of which have the same ability to append new Sup35 molecules. In this way, Hsp104 could complete the replication cycle of yeast prions, which is essential for prion inheritance. However, if there is too much of Hsp104 present, it could dissolve the prion fibrils entirely. Numerous confirmations for the major points of this model were obtained later, but one provided by Michael’s lab was probably the simplest. However, this simplicity required making some methodical inventions. First, we established the conditions, in which association of prions with other molecules is disrupted, while prion fibrils are left intact. This allowed us to develop several novel biochemical techniques, including electrophoretic separation of prion oligomers in agarose gel termed SDD-AGE (semi-denaturing detergent agarose gel electrophoresis), which became widely used afterwards [22]. This allowed to determine the size of prion units, which turned to be rather small, in the range of 10 to 50 Sup35 monomers per unit. When Hsp104 activity was inhibited by guanidine hydrochloride, Sup35 prion units began to grow in size almost twofold at each cell generation. This result was highly reproducible and corresponded to expectations in case if prion fragmentation would be blocked [23].

Later works of Michael’s lab addressed fine details of prion and amyloid formation in yeast. They found that Sup35 can form non-heritable amyloids, when overproduced in the presence of another prion, [*PIN*^+^] [24], an important phenomenon, the mechanism of which is still not fully understood. Study of the nature of ‘species’ barrier for prion propagation revealed that the barrier can occur due to the different mechanisms, depending on particular differences in prion protein sequence [25]. Two other works showed that particular amino acid residues are very different in their ability to elicit fragmentation of aggregated proteins, containing these sequences by Hsp104 [26,27]. Aromatic residues were most efficient in promoting fragmentation. Unexpectedly, fragmentation capabilities did not generally correlate with hydrophobicity. The interest in amyloids also resulted in studies of polyglutamine toxicity in the yeast model. These studies revealed that the amyloid polymerization of one protein can initiate a cascade of polymerization of several other amyloidogenic proteins, which in turn can sequester their non-amyloidogenic partners, thus interfering with their function [28]. Surprisingly, one such toxic cascade involved Q25-Htt, the huntingtin variant, which is usually used as a non-pathogenic control.

Despite his extensive work on prions, Michael retained an interest in the normal functions of Sup35 and Sup45. He showed that both proteins have functions besides translation, and these functions are also essential for cell viability. Some of these functions were related to actin cytoskeleton, cytokinesis and control of DNA replication [29,30]. Understanding the impact of these findings still awaits further investigation.

One of the reasons for the organization of a yeast laboratory in Cardiology Research Center was an anticipation of its participation in biotechnological projects employing yeast for the production of medically important proteins. This has led to emergence of an additional model organism in Michael’s lab, namely methylotrophic yeast *Hansenula polymorpha*. Using *Hansenula*, it was shown that a defect of the retrograde vesicular transport between the secretory organelles affects calcium homeostasis, probably due to the disruption of calcium delivery from the vacuole to the endoplasmic reticulum [31,32]. Michael’s team has also shown that, unlike *S. cerevisiae, H. polymorpha* lacking both Golgi apparatus Ca^2+^/Mn^2+^ ATPase Pmr1 and vacuolar Ca^2+^ ATPase Pmc1, dies not due to excess of Ca^2+^ in the cytosol but due to Ca^2+^ shortage in the secretory pathway [32]. The most recent study by Michael’s lab, involving *H. polymorpha* as a model organism revealed a link between protein glycosylation and phosphate transport [33].

Michael was a very democratic leader, and important questions of various kinds were usually decided at the laboratory seminars. To the labmates, he was more like a senior friend, ready to help in any difficult situation. And, of course, the first priority and important source of motivation in the lab was keeping science up to the highest quality standards. He would never allow publication of any data, of which he was not fully confident.

Goodbye, Misha. We will miss you very much, and will try to further develop the ideas originated from you.
